# Filter-Coupled Heteroscedastic Gaussian Process Regression for S–N Curve Prediction of Injection-Molded Short Glass-Fiber-Reinforced Thermoplastics

**DOI:** 10.3390/polym18212627

**Published:** 2026-10-23

**Authors:** Jorge Jiménez-Armendáriz, Mariel Alfaro-Ponce, Cristopher A. Muñoz-Ibañez, Moises Jimenez-Martinez

**Affiliations:** 1 School of Engineering and Sciences, Tecnologico de Monterrey, Via Atlixcayotl 5718, Puebla 72453, Mexico; a01327172@tec.mx; 2 Artificial Intelligence for Manufacturing and Supply Chain Institute, Calle del Puente 222 Col. Ejidos de Huipulco Tlalpan, Ciudad de Mexico 14380, Mexico; 3 School of Engineering and Sciences, Tecnologico de Monterrey, Blvd. Felipe Ángeles 2003, Venta Prieta, Pachuca de Soto 42080, Mexico; cristopher_antonio@tec.mx

**Keywords:** S–N curve, Gaussian process regression, uncertainty quantification, injection molding, short glass-fiber-reinforced thermoplastics, machine learning

## Abstract

Predicting the fatigue behavior of injection-moulded short glass-fiber-reinforced thermoplastics is hindered by the large, irreducible scatter of fatigue data and by the strong dependence of the response on the moulding conditions, which conventional point-estimate models neither reproduce nor quantify. This study presents a physics-informed heteroscedastic Gaussian process regression model (Pi-HGPR) that reconstructs the S–N curve of a given processing condition by predicting the fatigue stress amplitude as a function of the number of cycles, the injection temperature, the packing pressure, the specimen orientation, and the stress ratio, and that returns a 95% prediction band rather than a single value. The model combines a damage process-type physical mean, an anisotropic Matérn 5/2 covariance with Automatic Relevance Determination, and a local dispersion filter coupled to the process likelihood, which makes the noise level input-dependent. Trained on S–N curves compiled from the literature and assessed under two out-of-fold validation protocols (point-wise interpolation and leave-condition-out extrapolation), the model reaches a coefficient of determination of $R^{2}=0.839$ in interpolation and $R^{2}=0.649$ in leave-condition-out extrapolation, outperforming Random Forest and gradient-boosting baselines in both regimes, with a global empirical coverage of the prediction band of 92.9% and 86.2%, respectively, slightly below its nominal value and lower (76–81%) in sparsely sampled regions of the processing space. Automatic Relevance Determination identifies the packing pressure and the injection temperature as the most influential inputs, ahead of the stress ratio and the specimen orientation. Framing the task as predicting the stress amplitude along the low-scatter direction of the S–N field, and incorporating an input-dependent uncertainty, yields an accurate model within the tested processing window and provides reliable extrapolation limits governed by experimental data coverage rather than the learner.

## 1. Introduction

Injection molding is a high-volume manufacturing process that allows the production of complex geometries with high dimensional precision in reduced manufacturing time. The dimensional and geometrical quality of the injected part depends on the correct design of the cavity, feeding, and cooling systems. Process parameters like mold temperature, melt temperature, holding time, cooling time, injection rate, holding temperature, holding pressure, and injection pressure can be modified to obtain good dimensional stability and microstructure [1,2].

For injection-molded short glass fiber-reinforced composites, mechanical properties depend on matrix properties, fiber properties, strength of interface, and fiber orientation and distribution [3]. In the last 40 years, several approaches have been followed to increase the mechanical properties of injected glass fiber-reinforced polymers. First, Sanou et al. compared filled and unfilled injected polymers, finding that fibers are flow-oriented and the degree of orientation is affected by melt temperature and volumetric flow rate [4]. Later, Eggert et al. proposed a pp-based binder for better adhesion between fiber and matrix, increasing interface strength. This modification increases tensile and fracture properties [5]. Fiber length and interface strength can also be increased by modifying pelletizing parameters, like melt temperature and screw speed during the process [6]. Mechanical properties can also be increased by varying the fiber content by directly feeding fibers during plasticizing [7].

Regarding the effect of pressure and temperature changes in the part’s microstructure and resulting mechanical properties: in the polymeric matrix, solidification due to the temperature gradient between melt flow and outer walls [8] and orientation of polymeric chains are influenced by melt temperature and packing pressure [9]. These process parameters also determine the orientation and distribution of fibers in the matrix [10]. Liou et al. found that increasing packing pressure increases the number of oriented fibers, while increasing injection temperature from 230 °C to 250 °C increases tensile strength by promoting a uniform dispersion of fibers in the matrix [11].

Due to the numerous process parameters that influence the resulting mechanical properties, it is necessary to optimize the response with multiple dependent variables. Design of experiments, analytical models, and ANOVA have been implemented to study the effect of process parameters. Following a design of experiments, Lafranche et al. determined the effect of melt temperature on flexural properties of PA6GF and compared the relations found with microstructure analysis [12]. Dahrmann et al. followed a design of experiments resulting in 47 variants to determine the effect of screw speed on tensile, impact, and rheological properties of injection-molded PP, finding no significant influence of this parameter on the measured properties [13]. Following a DOE, Dupuis et al. found an effect of injection speed and packing pressure on crystallization of outer layers and on the concentration and orientation of fibers [14].

Determining the resulting fatigue properties of the different modifications and combinations of process parameters of glass fiber-reinforced polymers is critical. Although quasi-static mechanical properties can be improved, when submitted to alternating loads during fatigue, even below the limit, cracks form over time and propagate, resulting in complete failure of the material [15].

Conventional design of experiments (DoE) has been widely used to quantify how material and processing modifications—such as process parameters, fiber type and length, and interfacial strength—affect fatigue resistance in fiber-reinforced polymers. Prior studies report improved crack-growth resistance with increasing fiber volume fraction [16] and enhanced fatigue performance with longer fibers in glass fiber-reinforced polypropylene [17]. DoE approaches have also been applied to compare fillers, identifying talc and glass fiber as particularly effective in extending fatigue life [18]. In contrast, systematic investigations focused specifically on the influence of processing parameters on the fatigue resistance of glass fiber-reinforced polymers remain limited; related evidence in talc-filled polypropylene suggests that higher hold pressure and melt temperature can improve tensile and fatigue properties [19].

Fatigue modeling of composite materials is challenged by the large scatter in fatigue life observed even for specimens with identical constituents and nominal manufacturing conditions, which is commonly attributed to variations in fiber distribution and orientation, interfacial strength, and manufacturing defects [20]. As a result, conventional finite element analyses based on isotropic assumptions can yield significant prediction errors [21]. While explicit micro-mechanical modeling of fiber, matrix, and interface is feasible for long unidirectional composites, it is computationally demanding and requires detailed orientation information that is often unavailable for short fiber-reinforced systems [22]. Energy-based approaches have therefore been proposed to estimate fatigue life from damage evolution and crack nucleation during testing [23].

Despite these advances, assessing the impact of new material or process modifications still typically requires extensive experimental campaigns, and data-driven extrapolations remain limited by fatigue scatter. To improve fatigue-life prediction under uncertainty and multiple interacting failure mechanisms, artificial neural networks (ANN) have been used to learn relationships between measurable material/process descriptors (e.g., fiber type and content, matrix, and process parameters) and controllable loading conditions (e.g., stress ratio and load amplitude) and the resulting fatigue life [24,25]. Early ANN studies demonstrated promising predictions for glass fiber-reinforced composites under constant-stress fatigue but often neglected manufacturing conditions [26,27]. Subsequent work improved accuracy by incorporating quasi-static properties and/or orientation information [28,29], yet these models were largely developed for non-injection-molded composites or different material systems. Consequently, predicting S–N behavior across varying injection-molding processing conditions remains insufficiently addressed.

The inherent uncertainty in fatigue input data further limits prediction accuracy [30], motivating probabilistic, data-driven approaches for remaining useful life (RUL) estimation. Bayesian methods have been coupled with indirect damage-prognosis measurements—such as acoustic emission and conductivity/electrical resistance changes—to infer damage evolution and RUL. For example, Bayesian-regularized neural networks have been used to predict fatigue life and damage state of glass fiber-reinforced composites from resistance measurements, improving robustness to complex nonlinear relationships [31]. Bayesian neural networks trained with acoustic emission data have also enabled real-time RUL prediction while providing confidence intervals, addressing the deterministic nature of standard ANNs [32]. More recently, approximate Bayesian computation-based training has been proposed to quantify uncertainty in stiffness reduction and microcrack density evolution as an alternative to physics-based models [33]. In parallel, Gaussian process regression (GPR) has been applied to fatigue degradation monitoring using acoustic emission, offering uncertainty-aware RUL distributions and reduced training time compared with ANN [34].

Beyond prognosis, GPR-based models have been used to predict fatigue life under varying loading conditions and material descriptors, including multiaxial fatigue (via ANN–GPR hybrids) and structural components where stress state, material properties, and geometry are inputs [35,36]. However, the use of GPR to reconstruct the S–N curve from experimental fatigue data for the same material produced under different processing conditions remains largely unexplored.

In this work, a heteroscedastic Gaussian process regression (GPR) model is trained with fatigue data compiled from the literature for PPGF, PA6GF, and PBTGF, all reinforced with 30% short glass fiber. From each of the reviewed studies the injection temperature and packing pressure were identified, together with the specimen orientation θ (the angle between the specimen axis and the melt-flow direction), while the stress ratio R was taken as a loading variable and the number of cycles as the abscissa of the S–N curve; the test frequency was recorded but treated as an experimental-protocol variable and excluded from the model inputs. Rather than the fatigue life, the model predicts the fatigue stress amplitude *s*; that is, it reconstructs the S–N curve for each new combination of processing and loading parameters and returns not a single value but a predictive distribution: a mean estimate together with a 95% prediction band that reflects the inherent scatter of the fatigue data. The physically motivated mean of the process is complemented with a coupled, input-dependent noise model, so that the width of the band adapts to the local dispersion of the data. For each new combination of process parameters, under a particular load condition, the predicted curve is compared to the fitting curve of the actual data and its prediction interval, and the capacity of the model to interpolate within the tested processing window and to extrapolate to unseen processing combinations is discussed under two separate validation protocols.

### 1.1. Main Contribution

The main contribution of this work is a heteroscedastic Gaussian Process Regression model, coupled to a local noise filter and equipped with a damage model-type physical mean, that predicts the fatigue stress amplitude of injection-molded short glass-fiber-reinforced thermoplastics as a function of the number of cycles and the moulding and loading parameters, while natively quantifying the inherent scatter of fatigue through 95% prediction bands. The role of the coupled local filter is to make the width of the prediction band input-dependent, so that it follows the local dispersion of the data, rather than to increase the point accuracy of the mean prediction. In contrast to previous ANN approaches [25,26,27,28,29], which return point estimates and require the processing conditions to have been seen during training, the proposed model provides an input-dependent uncertainty that widens where the data are scarce or dispersed and narrows where the S–N response is well determined; it embeds a physically consistent mean so that predictions remain fatigue-consistent away from the training data; and it is assessed under two complementary protocols—point-wise interpolation and leave-condition-out extrapolation—that separate performance within the tested processing window from generalization to genuinely new processing combinations, exposing that the limit on extrapolation is set by the coverage of the experimental data rather than by the choice of learner.

### 1.2. Paper Organization

The remainder of this paper is organized as follows. Section 2 formulates the heteroscedastic Gaussian Process Regression model, introducing the physical mean function, the anisotropic Matérn 5/2 covariance with automatic relevance determination, the coupled local filter that supplies the input-dependent noise, and the predictive and hyperparameter-optimization scheme. Section 3 describes the fatigue database compiled from the literature, the selection criteria and the conditioning of the data. Section 4 presents and discusses the results: the comparison of algorithms under the two validation protocols, the ablation of the pipeline, the relevance analysis of the input variables, and the predictive uncertainty. Finally, Section 5 summarizes the main findings and outlines directions for future work.

## 2. Heteroscedastic Gaussian Process Regression Model Formulation

As previously stated, the fatigue stress amplitude was modeled with Gaussian process regression (GPR). A Gaussian process (GP) is a collection of random variables, any finite number of which have a joint Gaussian distribution, and is completely specified by its mean and covariance functions. It places a probability distribution directly over functions,

(1) $$f\left(x\right)∼\mathrm{GP}\;\left(m\left(x\right),\;k\left(x,x^{\prime }\right)\right),$$

where $x\in R^{D}$ is a *D*-dimensional input vector, m(x)=E[f(x)] is the mean function, and $k\left(x,x^{\prime }\right)=\mathrm{Cov}\left[f\left(x\right),f\left(x^{\prime }\right)\right]$ is the covariance (kernel) function.

In contrast to the customary homoscedastic assumption of a single noise variance, the observations $y_{i}$ are here assumed to be corrupted by *input-dependent* additive Gaussian noise,

(2) $$y_{i}=f\left(x_{i}\right)+\varepsilon_{i},\;\varepsilon_{i}∼N\;\left(0,\;\sigma_{n}^{2}\left(x_{i}\right)\right),$$

so that the noise variance $\sigma_{n}^{2}\left(x\right)$ is a function of the input rather than a constant. This is the central modeling choice of the present work. Fatigue scatter is not uniform: it depends on the stress level, on the number of cycles and on the processing condition, and runout (censored) observations carry a fundamentally larger uncertainty than recorded failures. A single global $\sigma_{n}^{2}$ must compromise between these regimes, either over-smoothing the low-scatter portions of the S–N field or under-representing the high-scatter ones. An input-dependent $\sigma_{n}^{2}\left(x\right)$, estimated by a local filter that is *coupled* to the GP fit (Section 2.4), yields a predictive band whose width tracks the local dispersion of the data and thus an input-dependent quantification of the inherent fatigue scatter.

### 2.1. Mean Function

In contrast to the customary zero prior mean m(x)=0, a parametric mean function is adopted, so that the covariance function models only the residual structure that the mean does not capture,

(3) $$m\left(x\right)=\beta_{0}+\beta_{1}\log_{10}N,$$

a log–linear S–N trend whose intercept $\beta_{0}$ and slope $\beta_{1}$ are estimated by ordinary least squares on the training partition. Because the cycle count enters as $\log_{10}N$ and the target as $\log_{10}s$ (Section 2.3), this mean captures the first-order S–N dependence of the stress amplitude on life, and the covariance is left to model only the processing-dependent departures from it. At a query far from the training data, the Gaussian process reverts to m(x), so a physically meaningful mean yields a fatigue-consistent prediction rather than the global average returned by a zero-mean prior.

### 2.2. Covariance Function

The covariance was chosen from the Matérn family with smoothness parameter ν=5/2, which yields functions that are twice mean-square differentiable—smooth enough to be physically plausible, yet less smooth than the squared-exponential, which suits noisy experimental data. Automatic relevance determination (ARD) was incorporated by assigning an independent length-scale $\ell_{d}$ to each of the *D* input dimensions, so that the kernel depends on the scaled distance *r*,

(4) $$k\left(x,x^{\prime }\right)=\sigma_{f}^{2}\left(1+\sqrt{5}\;r+\frac{5}{3}r^{2}\right)\exp\;\left(-\sqrt{5}\;r\right),\;r=\sqrt{\sum_{d=1}^{D}\frac{{\left(x_{d}-x_{d}^{\prime }\right)}^{2}}{\ell_{d}^{2}}},$$

or, equivalently, $r^{2}={\left(x-x^{\prime }\right)}^{⊤}\Lambda^{-1}\left(x-x^{\prime }\right)$ with $\Lambda =\mathrm{diag}(\ell_{1}^{2},\ldots ,\ell_{D}^{2})$. Here, $\sigma_{f}^{2}$ is the signal variance (the prior variance in the latent function *f*). The quantity $1/\ell_{d}$ measures the relevance of dimension *d*: a large $\ell_{d}$ renders variation in $x_{d}$ almost invisible to the covariance, so that the hyperparameter fit performs a soft, data-driven selection among the inputs.

### 2.3. Inputs and Desired Output

The regression target is the fatigue stress amplitude $s\equiv \sigma_{a}=\left(\sigma_{\max}-\sigma_{\min}\right)/2$, where $\sigma_{\max}$ and $\sigma_{\min}$ are the maximum and minimum stresses of the loading cycle (MPa), modeled as a function of the five-dimensional input vector,

(5) $$x={\left[\;\log_{10}N,\;T_{\mathrm{injection}},\;P_{\mathrm{packing}},\;\theta ,\;R\;\right]}^{⊤},\;D=5,$$

where *N* is the number of cycles to failure, $T_{\mathrm{injection}}$ is the melt injection temperature (°C), $P_{\mathrm{packing}}$ is the packing (holding) pressure (MPa), θ is the specimen orientation, i.e., the angle between the specimen axis and the melt-flow direction (°), and $R=\sigma_{\min}/\sigma_{\max}$ is the stress ratio (dimensionless). This vector jointly encodes the loading history, the molding process state and the microstructural anisotropy of the specimen. Prior to fitting, the target and the cycle count are mapped to a base-10 logarithmic scale ($y=\log_{10}s$, $\log_{10}N$) so that the multiplicative scatter characteristic of fatigue data becomes approximately additive; the inputs are then standardized to zero mean and unit variance so that the recovered length-scales $\ell_{d}$ are directly comparable across physically heterogeneous variables.

### 2.4. Coupled Heteroscedastic Noise Model

The input-dependent variance $\sigma_{n}^{2}\left(x\right)$ in Equation (2) is estimated by a local (Nadaraya–Watson) dispersion filter that is coupled to the GP through the likelihood. Let $r_{i}=y_{i}-m\left(x_{i}\right)-\bar{f}\left(x_{i}\right)$ denote the residual of observation *i* about the current mean and latent GP trend. For each point, its *k* nearest neighbors $N_{k}\left(i\right)$ in the standardized input space are identified, with squared distances $d_{ij}^{2}={∥x_{i}-x_{j}∥}^{2}$ and Gaussian weights

(6) $$w_{ij}=\frac{\exp\;\left(-d_{ij}^{2}/2h^{2}\right)}{\sum_{l\in N_{k}\left(i\right)}\exp\;\left(-d_{il}^{2}/2h^{2}\right)},\;h=\mathrm{median}\;d_{ij},$$

where the bandwidth *h* is set to the median neighbor distance. The filter returns a local trend and a local weighted dispersion,

(7) $$t_{i}=\sum_{j\in N_{k}\left(i\right)}w_{ij}\;r_{j},\;{\hat{\sigma }}^{2}\left(x_{i}\right)=\sum_{j\in N_{k}\left(i\right)}w_{ij}\;{\left(r_{j}-t_{i}\right)}^{2}.$$

Censored (runout) observations are treated as lower bounds on life by inflating their local variance, ${\hat{\sigma }}^{2}\left(x_{i}\right)\leftarrow c\;{\hat{\sigma }}^{2}\left(x_{i}\right)$ with c>1, so that they inform the latent mean only weakly and widen the band in the high-cycle regime. The estimates populate the diagonal noise matrix $\sum =\mathrm{diag}\;\left({\hat{\sigma }}^{2}\left(x_{1}\right),\ldots ,{\hat{\sigma }}^{2}\left(x_{n}\right)\right)$.

The filter and GP are fitted jointly by the alternating scheme of Algorithm 1, an expectation–maximization/iteratively-reweighted procedure: the filter supplies the noise field on which the GP conditions, and the GP supplies the smooth trend about which the filter measures dispersion. The two are iterated, with a relaxation factor λ∈(0,1] for stability, until the noise field converges.

This coupled filter supersedes the separate monotonic pre-filter of a preliminary homoscedastic formulation explored during the development of this study (Section 4.2). Rather than smoothing the within-curve scatter away—which improves interpolation but drives the noise level and, hence, the predictive band, towards zero—the filter *estimates* scatter and represents it as input-dependent uncertainty so that interpolation accuracy and a scatter-enveloping band are no longer in tension. If a shape-preserving prior is desired, monotonicity can be retained as a constraint on the local trend $t_{i}$ (enforcing $t_{i}$ non-increasing in $\log_{10}N$) without discarding the dispersion information.
**Algorithm 1** Filter–GP coupling1:Initialise $\sum^{\left(0\right)}={\hat{s}}^{2}I$                                           ▹${\hat{s}}^{2}$: variance in the mean residual2:**for** t=1,…,T **do**3:    Optimise kernel hyperparameters (Section 2.6) with $K_{y}=K+\sum^{\left(t-1\right)}$4:    Compute residuals $r_{i}=y_{i}-m\left(x_{i}\right)-\bar{f}\left(x_{i}\right)$5:    Estimate ${\hat{\sigma }}^{2}\left(x_{i}\right)$ from Equations (6) and (7); set $\sum^{\prime }=\mathrm{diag}\left({\hat{\sigma }}^{2}\right)$6:    Update $\sum^{\left(t\right)}=\left(1-\lambda \right)\;\sum^{\left(t-1\right)}+\lambda \;\sum^{\prime }$7:    **stop** if $∥\sum^{\left(t\right)}-\sum^{\left(t-1\right)}∥$ is below tolerance8:**end for**

### 2.5. Predictive Distribution

Given the training set (X,y), with Gram matrix ${\left[K\right]}_{ij}=k\left(x_{i},x_{j}\right)$, cross-covariance vector $k_{*}={\left[k\left(x_{*},x_{1}\right),\ldots ,k\left(x_{*},x_{n}\right)\right]}^{⊤}$, and heteroscedastic noise matrix ∑, the predictive distribution of the latent function at a new input $x_{*}$ is Gaussian with mean and variance

(8) $$\begin{array}{cc} {\bar{f}}_{*} & =m_{*}+k_{*}^{⊤}{\left(K+\sum \right)}^{-1}\left(y-m\right), \end{array}$$

(9) $$\begin{array}{cc} V[f_{*}] & =k\left(x_{*},x_{*}\right)-k_{*}^{⊤}{\left(K+\sum \right)}^{-1}k_{*}, \end{array}$$

where m and $m_{*}$ are the mean function evaluated at the training inputs and at $x_{*}$. The predictive variance of an *observed* amplitude adds the aleatoric term at the query point,

(10) $$\sigma^{2}\left(y_{*}\right)=V\left[f_{*}\right]+\sigma_{n}^{2}\left(x_{*}\right),$$

where $\sigma_{n}^{2}\left(x_{*}\right)$ is the local noise interpolated to $x_{*}$ as the weighted mean of ${\hat{\sigma }}^{2}$ over its neighbors. The 95% prediction band on *s* is obtained by the back-transformation of ${\bar{f}}_{*}\pm 1.96\;\sigma \left(y_{*}\right)$ from the logarithmic scale. Because Equation (10) includes the aleatoric noise at the query point, this band is a prediction interval for a new observation, not a confidence interval for the mean function; it is therefore referred to as a prediction band throughout. Because the noise term in Equation (10) is input-dependent, the band widens automatically in regions of high experimental scatter and around censored data, and narrows where the S–N response is well determined.

### 2.6. Hyperparameter Optimization

The kernel hyperparameters $\theta =\{\sigma_{f}^{2},\ell_{1},\ldots ,\ell_{D}\}$ were obtained in two stages, interleaved with the noise-field update of Algorithm 1.

Stage 1—marginal-likelihood optimization

For a fixed noise field ∑, the relative length-scales (the ARD shape) and the signal variance were obtained by maximizing the log marginal likelihood,

(11) $$\log p\left(y|X,\theta \right)=-\frac{1}{2}{\left(y-m\right)}^{⊤}K_{y}^{-1}\left(y-m\right)-\frac{1}{2}\log\left|K_{y}\right|-\frac{n}{2}\log2\pi ,\;K_{y}=K+\sum ,$$

whose first term rewards data fit and whose second penalises model complexity, embodying an automatic Occam’s razor trade-off [37,38]. The optimization is carried out on the logarithm of the hyperparameters with the limited-memory Broyden–Fletcher–Goldfarb–Shanno algorithm with bound constraints (L-BFGS-B) [39] and several random restarts, retaining the highest-likelihood solution. Stage 1 and the filter update alternate until convergence (Algorithm 1).

Stage 2—cross-validated length-scale magnitude

The marginal likelihood is a training-set criterion; maximizing it favors interpolation but provides no direct control over generalization to unseen conditions. A single isotropic scaling γ applied to all length-scales, $\ell_{d}\;\to \;\gamma \;\ell_{d}$, is therefore selected on an inner GroupKFold split of the training set. GroupKFold is a *k*-fold cross-validation scheme in which all the observations that share a group label—here, the same S–N curve—are assigned to the same fold [40], so that no curve contributes points to both the fitting and the validation folds; γ is chosen to minimize the leave-condition-out error, admitting smoother, more transferable solutions when the cross-validated error supports them. The selection is nested inside the outer evaluation, so that γ is never chosen on data used to score it.

## 3. Materials and Methods

### 3.1. Materials Database

Data for the database were collected from publications, surveyed from 1989 to 2024. These works were selected because the specimens tested to build the S–N curve obtained their final shape directly in the cavity or were cut from plates oriented to the melt flow; the fiber can then be considered oriented, and the anisotropy can be accounted for when specimens are cut with different orientations from the plates. Table 1 summarizes the process parameters and test conditions of the evaluated dataset.

Although axial-fatigue specimens can differ in shape among works, owing to the different standards used, the reported stress amplitude is used to compare S–N curves, as it is not affected by the cross-sectional area or shape of the specimen. S–N values belonging to specimens with notches or drilled holes on the faces were not considered, as these modifications significantly reduce the stress value. The reported stress values are not normalized by any of the mechanical properties reported in these publications; the stress directly applied on the specimen is used. Finally, for publications where tabular data were not available, the fatigue-life values were extracted by digitizing the published S–N plots using a custom Python 3.10 script based on Matplotlib 3.10 and NumPy 2.0. Data compiled for each material were obtained from tests run between 0.5 and 30 Hz. The loading frequency of each test was recorded but treated as an experimental-protocol variable rather than a design variable, and was therefore not used as a model input. Provided the cyclic self-heating remains below a temperature rise of about 10 °C, the S–N behavior of short glass-fiber-reinforced thermoplastics is essentially independent of the test frequency [57]; most of the compiled data were generated at 1–5 Hz, within this regime. Any residual frequency effect at the highest frequencies is not modeled as a mean trend but absorbed by the heteroscedastic noise term as predictive uncertainty (Section 4.6), and a robustness check confirms that excluding the points above 10 Hz leaves the reported performance essentially unchanged.

The three matrices considered are polypropylene (PPGF), polyamide-6 (PA6GF), and polybutylene terephthalate (PBTGF), all reinforced with 30% short glass fiber. When a specific process parameter was not reported by the author, the missing value was assigned from the process parameters recommended by the material manufacturer and the specifications of the injection-molding machine used in that work. The publications from which the process parameters and the corresponding S–N data were taken, together with the range of each variable, are summarized in Table 2.

### 3.2. Data Conditioning and Problem Definition

The compiled file contains 675 records; 5 exact duplicates were removed, leaving n=675 observations grouped into 81 distinct S–N curves (each curve being a unique combination of matrix, processing parameters, stress ratio, and specimen orientation), with a median of 5 points per curve. The fatigue life spans seven orders of magnitude (*N* from 1 to $1.1\times 10^{7}$ cycles), and the stress amplitude ranges from 7 to 180 MPa.

In contrast to the conventional formulation, in which the fatigue life is the output, here the problem is posed as the reconstruction of the S–N curve: the model predicts the fatigue stress amplitude *s* as a function of the number of cycles and the processing and loading state. This choice is deliberate. The fatigue life is the high-scatter direction of the S–N field—replicate specimens differ by a factor of 1.65 in *N* at fixed stress (an irreducible dispersion of 0.218 decades in $\log_{10}N$)—whereas the stress amplitude at fixed life is a low-scatter target, with an irreducible dispersion of only 0.036 decades in $\log_{10}s$ measured on exact replicates. Predicting *s* is therefore an intrinsically better-posed regression and generalises more reliably to unseen processing conditions. When the life associated with a given stress amplitude is required, it can be obtained by numerically inverting the predicted S–N curve, and the prediction band then provides the corresponding life interval.

The five inputs are the base-10 logarithm of the number of cycles $\log_{10}N$, the injection temperature $T_{\mathrm{injection}}$, the packing pressure $P_{\mathrm{packing}}$, the specimen orientation θ, and the stress ratio *R* (Equation (5)); the target is $\log_{10}s$. Both *N* and *s* enter on a logarithmic scale so that the multiplicative scatter characteristic of fatigue becomes approximately additive. The stress ratio takes three levels (R∈{−1,0.1,0.3}) and the orientation four ({0,30,45,90}°); both are treated as continuous numerical inputs taking their physical values. Encoding the orientation by its value in degrees, rather than by its rank, preserves the non-uniform spacing of the tested angles: standardization is an affine transformation so that $30^{\circ }$ remains closer to $45^{\circ }$ than to $0^{\circ }$ or $90^{\circ }$ in the input space, as in the physical angle. The choice is physically motivated, since the off-axis static and fatigue strength of short-fiber composites decreases monotonically and smoothly from the flow direction ($0^{\circ }$) to the transverse direction ($90^{\circ }$) [49,51], a smooth dependence that the Matérn covariance can interpolate. Prior to fitting, the inputs are standardized to zero mean and unit variance, with the scaler fitted on the training partition only, so that the recovered ARD length-scales are directly comparable across physically heterogeneous variables (Section 2). Runout specimens (those that did not fail, taken here as $N\geq 10^{6}$ cycles; 37 points, 5% of the data) are retained but flagged, and are treated as lower bounds on life through an inflated local noise in the heteroscedastic model (Section 2.4) rather than as exact observations.

The three matrices (PPGF, PA6GF, and PBTGF) are pooled into a single regression, and the matrix chemistry is not supplied as an explicit input. This is a deliberate scoping choice: all three share the same reinforcement (30% short glass fiber) and the target is the *processing-driven* departure from a common trend, not the absolute S–N level of a given polymer. The consequence is that the model separates materials only through their processing windows (Table 2), which partially overlap, and any systematic between-matrix offset in the S–N intercept is absorbed by the covariance rather than by the mean. This is one of the factors that bounds the extrapolation score reported in Section 4, and it is revisited as a limitation in Section 5.

### 3.3. General Pipeline and Reproducibility

The complete workflow, from the raw database to the reported indicators, is summarized in Algorithm 2 and designed to be fully reproducible. The model is the physics-informed heteroscedastic Gaussian process regression of Section 2: a Basquin-type linear mean in $\log_{10}N$,

(12) $$m\left(x\right)=1.937-0.079\;\log_{10}N,$$

fitted by ordinary least squares (its residual, with a standard deviation of 0.266 decades, is what the Gaussian process models), combined with an anisotropic Matérn 5/2 covariance with automatic relevance determination (one length-scale per input, bounded to $\left[10^{-2},10^{4}\right]$) and an input-dependent noise supplied by a coupled local filter. The filter estimates the local dispersion from the k=12 nearest neighbors in the standardized input space with Gaussian weights; runout points receive a six-fold inflation of their local variance; the filter and the Gaussian process are iterated T=4 times with a relaxation factor of 0.5 on the noise field, the kernel hyperparameters being re-optimized by marginal-likelihood maximization (L-BFGS-B) at each iteration. All stochastic components use a fixed seed (random_state = 42).
**Algorithm 2** General pipeline (target $\log_{10}s$, five inputs)  1:**Input:** fatigue database (675 records)  2:Remove exact duplicates → n=675 observations; assign a curve identifier to each of the 81 unique S–N conditions  3:Build inputs $x=[\log_{10}N,\;T_{\mathrm{injection}},\;P_{\mathrm{packing}},\;\theta ,\;R]$ and target $y=\log_{10}s$; flag runouts ($N\geq 10^{6}$)  4:**for** each evaluation protocol **do**  5:    **Interpolation:** 5-fold *K*-fold over the 675 points (shuffled, seed 42)  6:    **Extrapolation:** 5-fold *GroupKFold* over the 81 curves (leave-condition-out; ≈540 train/135 test points and ≈93/23 curves per fold)  7:    **for** each fold **do**  8:        Fit StandardScaler and the Basquin mean on the training set  9:        Fit the coupled heteroscedastic GPR (Section 2)10:        Predict mean and standard deviation on the held-out set11:    **end for**12:    Aggregate out-of-fold predictions and compute the indicators13:**end for**14:**Output:**$R^{2}$, RMSE and MAE on $\log_{10}s$, MAPE on *s*, fraction of predictions within ±10% and ±20% of *s*, and the empirical coverage of the 95% band

The same workflow is shown graphically in Figure 1.

The model is assessed under two complementary out-of-fold protocols that isolate two distinct engineering questions. The *interpolation* protocol uses a random 5-fold split over the 675 points and answers how well the model completes the S–N curve of a processing condition that has already been characterized. In this protocol, points of the same S–N curve appear by design in both the training and the test folds; it therefore measures the ability to complete a partially characterized curve and does not demonstrate generalization to an unseen curve, which is assessed only by the extrapolation protocol. The *extrapolation* protocol uses a 5-fold split *by curve* (leave-one-out): every fold removes entire S–N curves from training, so no point of a test curve is ever seen during training, and it answers how well the model predicts the S–N response of a genuinely new processing combination. The by-curve split is essential: a naive random split would place points of the same curve in both training and test sets and would therefore overestimate the accuracy attainable for a new processing combination. Model quality is reported not only through $R^{2}$ but, following common practice in fatigue, through the RMSE and MAE on $\log_{10}s$, the mean absolute percentage error on *s*, the fraction of predictions falling within ±10% and ±20% of the measured stress, and the empirical coverage of the nominal 95% prediction band, which quantifies whether the reported uncertainty is calibrated.

Model quality is reported through several complementary indicators, each answering a different practical question. The coefficient of determination $R^{2}$ measures the fraction of the observed variance in $\log_{10}s$ that the model reproduces: $R^{2}=1$ is a perfect fit and $R^{2}=0$ is no better than always predicting the mean, so higher is better. The root-mean-square error (RMSE) and the mean absolute error (MAE) quantify the typical magnitude of the prediction error, both expressed in decades of $\log_{10}s$ (one decade is a factor of ten in stress); they differ in that RMSE penalises large errors more heavily, so RMSE > MAE signals a few outlying predictions. Because an error in decades is not intuitive for design, the same error is also given as the mean absolute percentage error (MAPE) on the stress amplitude *s* itself, together with the fraction of predictions falling within ±10% and ±20% of the measured stress. Finally, the empirical coverage of the nominal 95% prediction band is the percentage of test points whose measured stress actually falls inside the predicted band; a well-calibrated model returns a coverage close to 95%, whereas a value far below it means the reported uncertainty is too narrow (over-confident) and far above it means it is too wide (over-conservative).

Point accuracy is reported on two scales for two audiences: on $\log_{10}s$, the scale on which the model is fitted and on which the scatter is approximately symmetric ($R^{2}$, RMSE, MAE), and after back-transformation to the stress amplitude *s*, as percentage errors (MAPE, ±10%/±20%), which are the quantities a designer reads directly. All percentage errors and the prediction band are computed on *s* after the predictions have been mapped back from $\log_{10}s$ to *s* by $s=10^{\;\hat{\log_{10}s}}$.

## 4. Results and Discussion

The model was evaluated under the two out-of-fold protocols defined in Section 3.3: point-wise interpolation (5-fold over the 675 points) and leave-condition-out extrapolation (5-fold by S–N curve). Unless stated otherwise, all figures reported below are out-of-fold, so that no point used to score a prediction was seen during training. In contrast to a preliminary formulation explored during the development of this study (Section 4.2), no S–N curve was removed from the dataset; the complete set of 81 curves is retained, and the robustness to dispersed conditions is handled through the model rather than by discarding data.

### 4.1. Comparison of Learning Algorithms

Table 3 compares the proposed physics-informed heteroscedastic GPR (Pi-HGPR) with two strong tree-based baselines, Random Forest and Histogram Gradient Boosting, all trained on the same five inputs to predict $\log_{10}s$. Pi-HGPR is the most accurate model in *both* regimes: it attains the highest interpolation $R^{2}$ (0.839 versus 0.790 and 0.788) and the lowest interpolation error (15.7% MAPE on *s*), and, more importantly, it also leads in extrapolation ($R^{2}=0.649$ versus 0.521 and 0.573). The tree ensembles are penalised in extrapolation because they revert to piece-wise-constant predictions outside the training support, whereas the Basquin mean of Pi-HGPR returns a physically consistent S–N trend for unseen conditions. Beyond point accuracy, and unlike the baselines, Pi-HGPR natively delivers a predictive distribution (Section 4.3), which is the quantity of interest for reliability-based design.

### 4.2. Ablation of the Pipeline

Table 4 reports the contribution of each component of the pipeline, added cumulatively over a plain homoscedastic zero-mean GPR. Two observations stand out. First, the Basquin mean function is the component with the largest effect: it raises the extrapolation score from $R^{2}=0.489$ to 0.643 and also slightly improves interpolation (0.841→0.855), confirming that anchoring the process to a physical S–N trend helps precisely where data are absent. Second, the coupled heteroscedastic filter leaves the point accuracy essentially unchanged ($R^{2}=0.839$/0.649 for interpolation/extrapolation), slightly reduces the extrapolation error (MAPE 29.9→28.5%), and keeps the global coverage of the 95% band at a similar level to that of the homoscedastic model (92→93% in interpolation, 89→86% in extrapolation). The contribution of the filter is therefore not an increase of the point accuracy or of the global coverage, but an input-dependent band whose width follows the local dispersion of the data, narrowing where the S–N response is well determined and widening around dispersed conditions and runouts. The slight decrease in the interpolation $R^{2}$ (from 0.855 to 0.839) is the expected consequence of this design: observations in high-dispersion regions receive a larger noise variance and therefore pull the latent mean less strongly, so that the model follows individual noisy points of already characterized curves slightly less closely. In extrapolation, the global coverage of the heteroscedastic band is slightly lower than that of the homoscedastic one because the local filter estimates the scatter from neighbouring training points and therefore underestimates the between-material variability when those neighbors belong to other materials (Section 4.3).

#### Comparison with a Preliminary Pre-Filtered Formulation

It is instructive to contrast these results with those obtained with a preliminary formulation explored by the authors during the development of this work, in which a homoscedastic GPR was combined with a separate monotonic pre-filter and the most dispersed S–N curves were removed from the dataset. That model reached a very high interpolation score ($R^{2}=0.976$, MAPE 4.9%) but an extrapolation score of only $R^{2}=0.263$, i.e., the pre-filter step that maximized interpolation simultaneously degraded extrapolation (which had peaked at 0.514 after the length-scale optimization). The reason is that the monotonic pre-filter *denoises the target before evaluation*: the model is then scored against a smoothed S–N curve rather than against the raw experimental scatter, which inflates the interpolation $R^{2}$ and deflates the MAPE. The present pipeline deliberately abandons that step. It predicts the raw stress amplitude and represents the fatigue scatter as input-dependent uncertainty instead of smoothing it away. As a consequence, the interpolation MAPE rises to a more honest 15.7%—an error measured on unsmoothed data—while the extrapolation $R^{2}$ improves from 0.263 to 0.649, more than doubling, and the predictive band reflects the experimental scatter instead of collapsing towards zero. The apparent loss of interpolation accuracy is therefore not a regression but the direct, and intended, consequence of no longer concealing the inherent dispersion of the data.

### 4.3. Overall Predictive Performance and Uncertainty Calibration

Table 5 collects the complete set of indicators for the final model. In interpolation, the model reconstructs the S–N curve of an already characterized processing condition with a typical error of 0.061 decades in $\log_{10}s$ (MAE), placing 84% of the predictions within ±20% of the measured stress (Figure 2). In extrapolation to genuinely unseen processing combinations, the error grows, as expected for a strict leave-condition-out protocol, yet the model still explains 65% of the variance and keeps 53% of its predictions within ±20% of the true stress. Of all held-out points, 92.9% (interpolation) and 86.2% (extrapolation) fall inside the nominal 95% prediction band, i.e., the band is close to calibrated when completing characterized curves and moderately over-confident when predicting unseen processing combinations. This is a global value; as shown below (Table 6), the coverage falls further in some sparsely sampled regions of the processing space. A model that both predicts and knows the confidence of its own prediction is more valuable for design than one with a higher point score and no uncertainty, because the band widens automatically around scarce or dispersed conditions and thereby signals when a new combination falls outside the region the data can support, although in the sparsest regions it does not widen enough to reach the nominal coverage. The distribution of the error across the 81 individual S–N curves is shown in Figure 3: 71% and 49% of the curves are predicted within 0.1 decades (≈±26% in stress) for interpolation and extrapolation, respectively. Figure 4 shows the predicted S–N curves (blue) with 95% prediction band for the best-, median- and worst-case conditions under each protocol; the median case represents the typical behavior of the model. The practical reading of these bands is that of a diagnostic: a narrow band indicates that the predicted curve can be used directly as a design allowable, whereas a wide band, as in the worst extrapolation cases, signals that the requested combination lies outside the region supported by the data, so that the prediction should not be used as a design limit and a confirmatory fatigue test is required.

To determine whether the errors are concentrated in specific materials or processing ranges, the out-of-fold predictions were disaggregated by specimen orientation, injection temperature and packing pressure (Table 6). In interpolation, the error remains moderate in all groups (MAPE between 5% and 26%), and the coverage of the prediction band stays between 90% and 100%. In extrapolation, the error concentrates in three regions: specimens tested along the flow direction ($\theta =0^{\circ }$; $R^{2}=0.42$, MAPE 34%), which gather the largest number of curves and the widest variety of materials; the highest packing pressures ($P_{\mathrm{packing}}>75$ MPa; MAPE 32%); the highest injection temperatures ($T_{\mathrm{injection}}>280$ °C; MAPE 33%, $R^{2}=0.04$), a region populated by few sources. Conversely, the $45^{\circ }$ conditions are predicted with a MAPE below 9% in both protocols (the $30^{\circ }$ group shows similar errors but contains only two curves, so its figures are merely indicative), and the intermediate packing pressures (46–75 MPa) with a MAPE of 19% and full coverage in extrapolation. The largest individual errors correspond to materials represented by one to three short S–N curves, for which no curve of the same material and similar processing exists in the training folds. In extrapolation, the coverage of the prediction band also falls below its nominal value in several groups, most markedly for $\theta =0^{\circ }$ (78%), $P_{\mathrm{packing}}\leq 45$ MPa (76%), and $T_{\mathrm{injection}}\leq 250$ °C (81%). Since the local filter estimates the scatter from the neighboring training points, it underestimates the between-material variability when those neighbours belong to other materials; the global coverage of 86% reported in Table 5 should therefore be read as an average, and the band of an untested condition in a sparsely sampled region should be interpreted with caution. These results confirm that the extrapolation error is governed by the coverage of the data in material and processing space rather than by a systematic bias of the model.

### 4.4. Relevance of the Process Parameters

The automatic relevance determination length-scales of the final kernel (Table 7) provide an interpretable ranking of the inputs: a short length-scale denotes a variable to which the fatigue response is highly sensitive. The packing pressure (ℓ=0.49) and the injection temperature (ℓ=0.58) are by far the most relevant inputs, with length-scales four to six times shorter than those of the stress ratio (ℓ=2.18) and the orientation (ℓ=3.20). This is consistent with the experimental literature, in which packing pressure controls the number of oriented fibers and injection temperature governs their dispersion in the matrix [11], and it provides direct, actionable guidance for process optimization. The number of cycles has the largest length-scale (ℓ=8.21), i.e., the smallest residual relevance, precisely because the Basquin mean function has already absorbed the first-order dependence of the stress amplitude on $\log_{10}N$, leaving the covariance to model only the processing-dependent departures from that trend.

### 4.5. Does Richer Feature Information Raise the Extrapolation Ceiling?

A natural question is whether the leave-one-out score is limited by the five-input representation. To test this, the inputs were augmented with the material descriptors already available in the database: Young’s modulus *E*, ultimate tensile strength UTS, strain at UTS, and $\log_{10}$(frequency), and the model was re-evaluated under two protocols of increasing severity. Under the leave-*curve*-out protocol, the augmented model appears to improve (mean $R^{2}$ over five shuffled fold assignments rising from 0.562±0.085 to 0.607±0.029). However, with only 40 distinct materials across 81 curves, a held-out curve usually shares its material with a training curve at different processing, so this gain may reflect recognition of material “siblings” rather than genuine generalization. A stricter leave-*material*-out protocol, in which every curve of a material is withheld simultaneously, removes this effect: there the augmented model scores $R^{2}=0.489$, *below* the five-input model’s 0.510. The additional descriptors therefore do not transfer to genuinely unseen materials—their values lie outside the training range—and marginally degrade performance. This confirms that the extrapolation ceiling is set by the coverage of the experimental data in material space, not by the learner or the input representation, and it justifies retaining the parsimonious five-input model.

### 4.6. Discussion

For a designer, an S–N model is never used as a single line but as a *design allowable*: a curve at a chosen survival probability (mean −kσ), which by definition requires the scatter band and not only the mean. This is precisely what the present model returns: a mean S–N response and a 95% prediction interval, so its output maps directly onto the statistical S–N allowables already used in fatigue design, obtained here from the processing parameters instead of from a dedicated test campaign per condition.

The quantity the model predicts, the stress amplitude, is also the well-conditioned one for design. Sizing a part fixes a target life and asks for the admissible stress; predicting stress answers that question directly. Reading the same curve in the life direction is intrinsically ill-conditioned for these materials: the fitted S–N slope is shallow ($d\log_{10}s/d\log_{10}N\approx -0.08$), so any error in stress is amplified by a factor of about 13 when expressed as a life. A method that predicts life must therefore fight this amplification, which is the deeper reason why life-oriented models only reach high scores under idealized validation (Section 4.6); predicting the stress allowable avoids it altogether.

On this basis, the reported figures are strong rather than weak. The interpolation error of 0.061 decades in $\log_{10}s$ corresponds to ±15% in stress, only about 1.8× the *irreducible* experimental scatter of the material itself (±8.6%, measured on exact replicates), and the ±20% stress band of the same order as the scatter bands routinely applied to fatigue allowables encloses 84% of the predictions. In extrapolation to an untested processing condition the stress error grows to ≈29%; whether this is acceptable is a design decision, but the model states it explicitly through its prediction band rather than leaving it to an assumed safety factor (an error expressed in decades of $\log_{10}s$ converts to a multiplicative factor in stress as $10^{\;\mathrm{error}}$: an error of 0.061 decades yields $10^{0.061}\approx 1.15$, i.e., about ±15% in *s*; likewise, the irreducible scatter of 0.036 decades yields $10^{0.036}\approx 1.086$, i.e., ±8.6%.).

The applicability of these results to actual injection-moulded parts must be qualified. The model is trained on specimen-level data compiled from the literature, and the molding parameters act as proxies for the microstructure they induce: fiber length distribution, fiber orientation and dispersion, matrix crystallinity, voids and manufacturing defects, and fiber–matrix interface quality. These variables are the physical mechanisms through which processing controls fatigue, but they are not reported systematically across the compiled sources and, when reported, they are measured with heterogeneous techniques (micro-computed tomography, optical microscopy, differential scanning calorimetry, and scanning electron microscopy), so they could not be used as consistent model inputs without discarding most of the database. As a consequence, two specimens moulded with the same nominal parameters but with different microstructures (e.g., owing to a different gate location and wall thickness or molding machine) are indistinguishable for the model, and this unresolved variability is absorbed by the heteroscedastic noise term as predictive uncertainty. The predictions therefore apply to regions of a part whose local microstructure is similar to that of the test specimens; for complex parts, the local fiber orientation predicted by mold-filling simulation would have to be mapped onto the orientation input, and thickness, weld-line, and notch effects are outside the scope of the present model.

#### Comparison with Previous Machine-Learning Approaches

Reported accuracies for machine-learning fatigue models of composites are often very high coefficients of determination between 0.85 and 0.99, but almost always under evaluation protocols that share information between training and test. Studies based on synthetically generated data [20], and life models augmented with Monte-Carlo data report $R^{2}$ up to 0.99 [60]; in both cases, points of the same S–N curve appear on both sides of the split, so the model interpolates within curves it has already partly seen. When the evaluation is made genuinely, predictive leave-one-out or leave-cluster-out over whole specimens or conditions, the same class of models degrades sharply, in some reports to $R^{2}\leq 0.1$ [61], and domain-knowledge features stop helping [62]. Earlier neural-network models for reinforced-polymer fatigue that did reach low error either used hand lay-up rather than injection molding and took the quasi-static properties as inputs [28], or addressed a different material system [29], and none of them reconstructs the S–N curve of an *unseen processing condition* from the molding parameters, nor reports a predictive uncertainty.

Seen in this light, the present results are competitive, not modest. Under a strict leave-one-out protocol, the honest analogue of predicting a new processing combination, the model retains $R^{2}=0.649$ and outperforms Random Forest and Gradient Boosting on the same data (Table 3), while its interpolation figures ($R^{2}=0.839$, ±15% in stress) are reported separately as a measure of curve completion and are not used as evidence of generalization, since, as in the literature, points of the same curve appear on both sides of a random split. The contribution is thus not only an accuracy number but a realistic estimate, based on a by-curve validation protocol, of how far processing-parameter-based fatigue prediction can be trusted, together with a model that reports its own confidence.

### 4.7. Practical Impact

Characterizing the S–N curve of a single new processing condition experimentally requires a full fatigue campaign, typically dozens of specimens over weeks, and the processing window spanned by injection temperature, packing pressure, orientation, and stress ratio is combinatorially too large to be explored exhaustively. Within the tested window, the proposed model returns the S–N curve and its design band for a new combination immediately, turning a multi-week campaign into a design-time query. Equally important, where a requested combination lies outside the region the data can support, the model does not fail silently: its band widens (although, in the sparsest regions, not always enough to reach the nominal coverage; Table 6), flagging that a confirmatory test is needed and, in effect, pointing to the single most informative experiment to run next. This reframes data-driven fatigue prediction from a black-box point estimator, which mechanical designers rightly distrust, into an uncertainty-aware tool that speaks the language of design allowables, and it is this combination of curve-wise validation, physical grounding and input-dependent uncertainty, rather than any single metric, that constitutes the main practical advance of this work.

## 5. Conclusions

This work presented a physics-informed heteroscedastic Gaussian process regression model (Pi-HGPR) that reconstructs the S–N curve of injection-molded short glass-fiber-reinforced thermoplastics (PPGF, PA6GF, and PBTGF) from the injection temperature, the packing pressure, the specimen orientation, and the stress ratio, and that returns a 95% prediction band together with the mean curve. The main conclusions are the following:

1. **Accuracy.** Trained on 675 points and 81 S–N curves, the model reaches $R^{2}=0.839$ (MAPE 15.7%; 84% of the predictions within ±20%) when completing already characterized curves, and $R^{2}=0.649$ (MAPE 28.5%) when predicting unseen processing combinations under leave-condition-out validation, outperforming Random Forest ($R^{2}=0.790/0.521$) and Histogram Gradient Boosting ($R^{2}=0.788/0.573$) in both regimes. Because the extrapolation score depends on how the curves are assigned to folds, its mean over five-fold assignments is 0.562±0.085.
2. **Uncertainty.** The coupled heteroscedastic filter does not increase the point accuracy; it makes the width of the prediction band input-dependent. The global empirical coverage of the band is 92.9% in interpolation and 86.2% in extrapolation, slightly below the nominal 95%, and drops to 76–81% in sparsely sampled processing regions, where the local filter underestimates the between-material variability.
3. **Physical consistency.** The Basquin-type mean is the most influential component of the pipeline, raising the extrapolation score from $R^{2}=0.489$ to 0.643. Predicting the stress amplitude instead of the life avoids the approximately thirteen-fold error amplification caused by the shallow S–N slope (≈−0.08), and the interpolation error (±15% in stress) is only about 1.8 times the irreducible experimental scatter (±8.6%).
4. **Relevance of the inputs.** Automatic relevance determination identifies the packing pressure (ℓ=0.49) and the injection temperature (ℓ=0.58) as the most influential inputs, ahead of the stress ratio and the specimen orientation.
5. **Limitations.** The extrapolation ceiling is set by the coverage of the data in material space: adding material descriptors does not improve the leave-material-out prediction ($R^{2}=0.489$ versus 0.510). The matrix is not an explicit input, microstructural variables (fiber length, orientation distribution, crystallinity, and voids) are not available consistently in the sources, and runouts are treated approximately through an inflated noise.

Future work will widen the processing and material coverage of the database, incorporate microstructural descriptors and a censored (Tobit-type) likelihood for runouts, and use the prediction band to select new experiments by active learning.

## Figures and Tables

**Figure 1:**
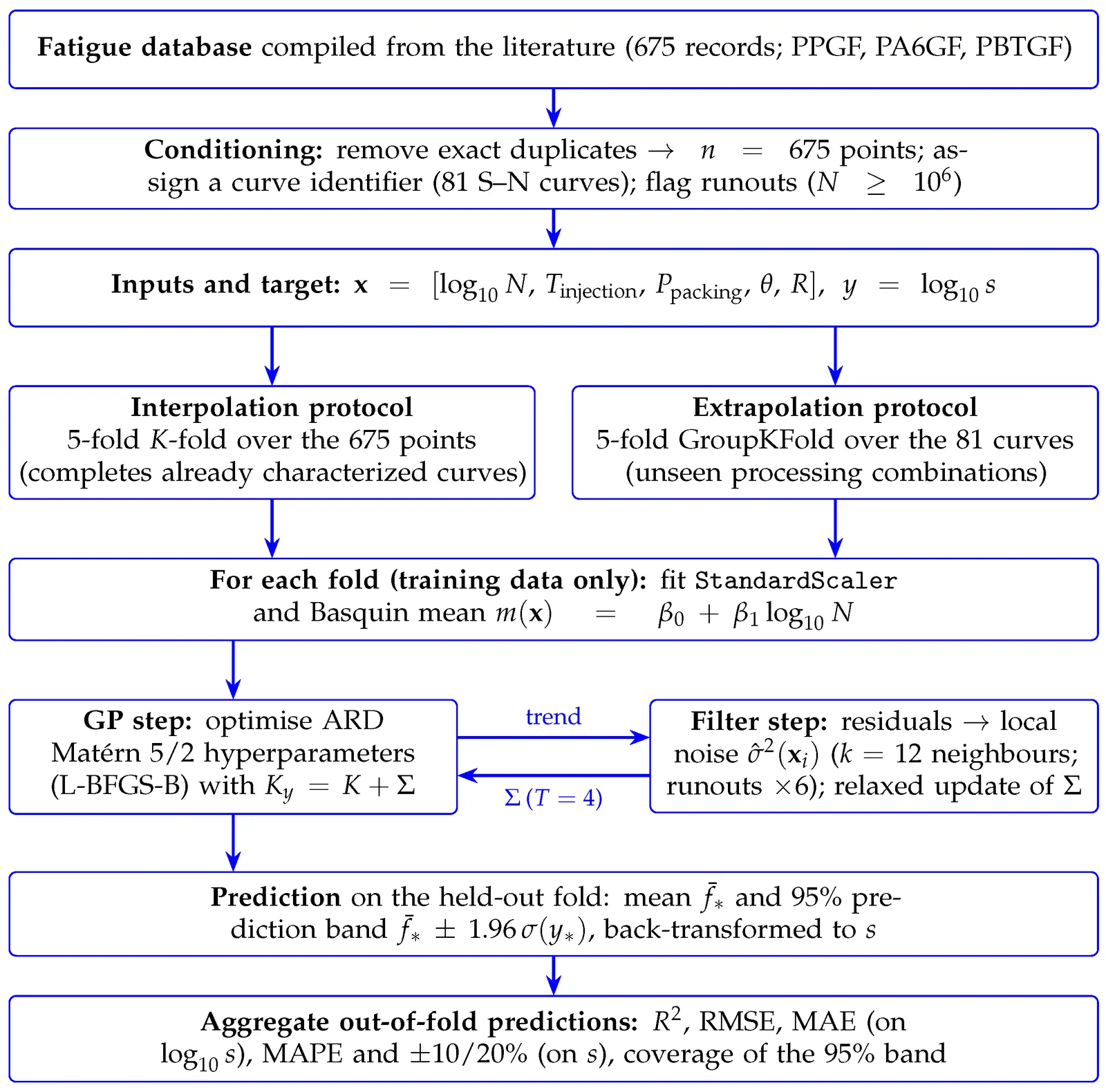
Graphical representation of the general pipeline (Algorithm 2) and of the filter–GP coupling (Algorithm 1). The two validation protocols share the same model-fitting steps and differ only in how the data are split into folds.

**Figure 2:**
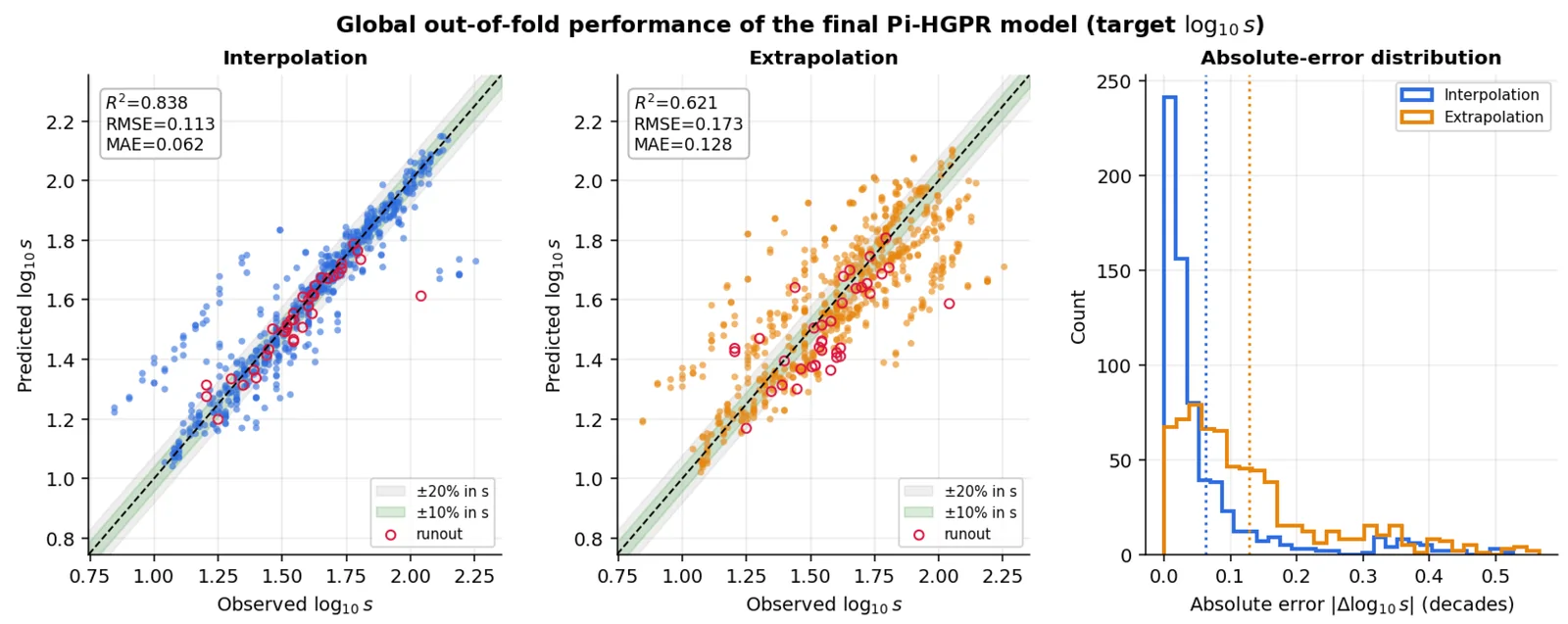
Global out-of-fold performance of the final Pi-HGPR model (target $\log_{10}s$). (**Left** and **center**): predicted vs. observed stress amplitude for interpolation and leave-condition-out extrapolation, with the ±10% and ±20% bands in *s* and the $R^{2}$, RMSE and MAE annotated; open red circles are runouts. (**Right**): distribution of the absolute error (dotted lines mark the mean).

**Figure 3:**
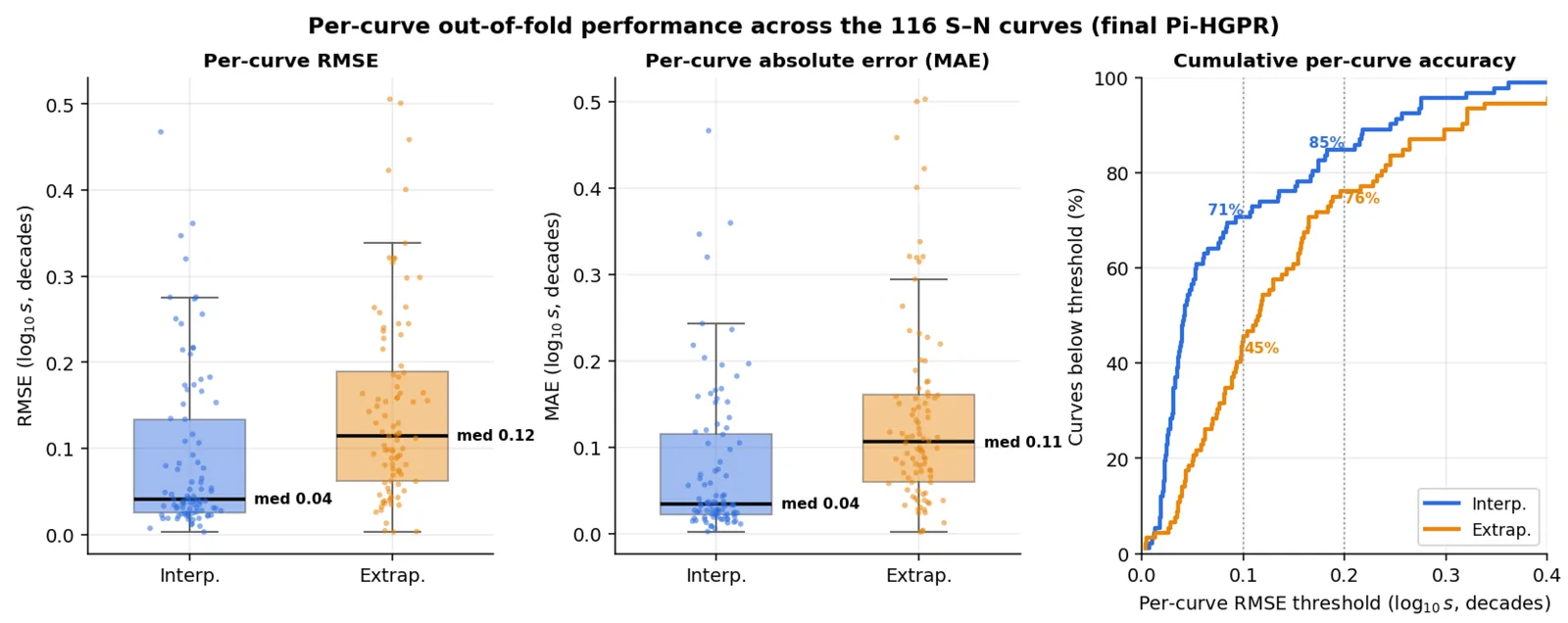
Per-curve out-of-fold performance across the 81 S–N curves. Each point is one curve. (**Left** and **center**): distribution of the per-curve RMSE and mean absolute error (MAE) in $\log_{10}s$ for interpolation and extrapolation. (**Right**): cumulative fraction of curves predicted below a given per-curve RMSE; 71%/49% of the curves fall within 0.1 decades and 84%/79% within 0.2 decades for interpolation/extrapolation, respectively.

**Figure 4:**
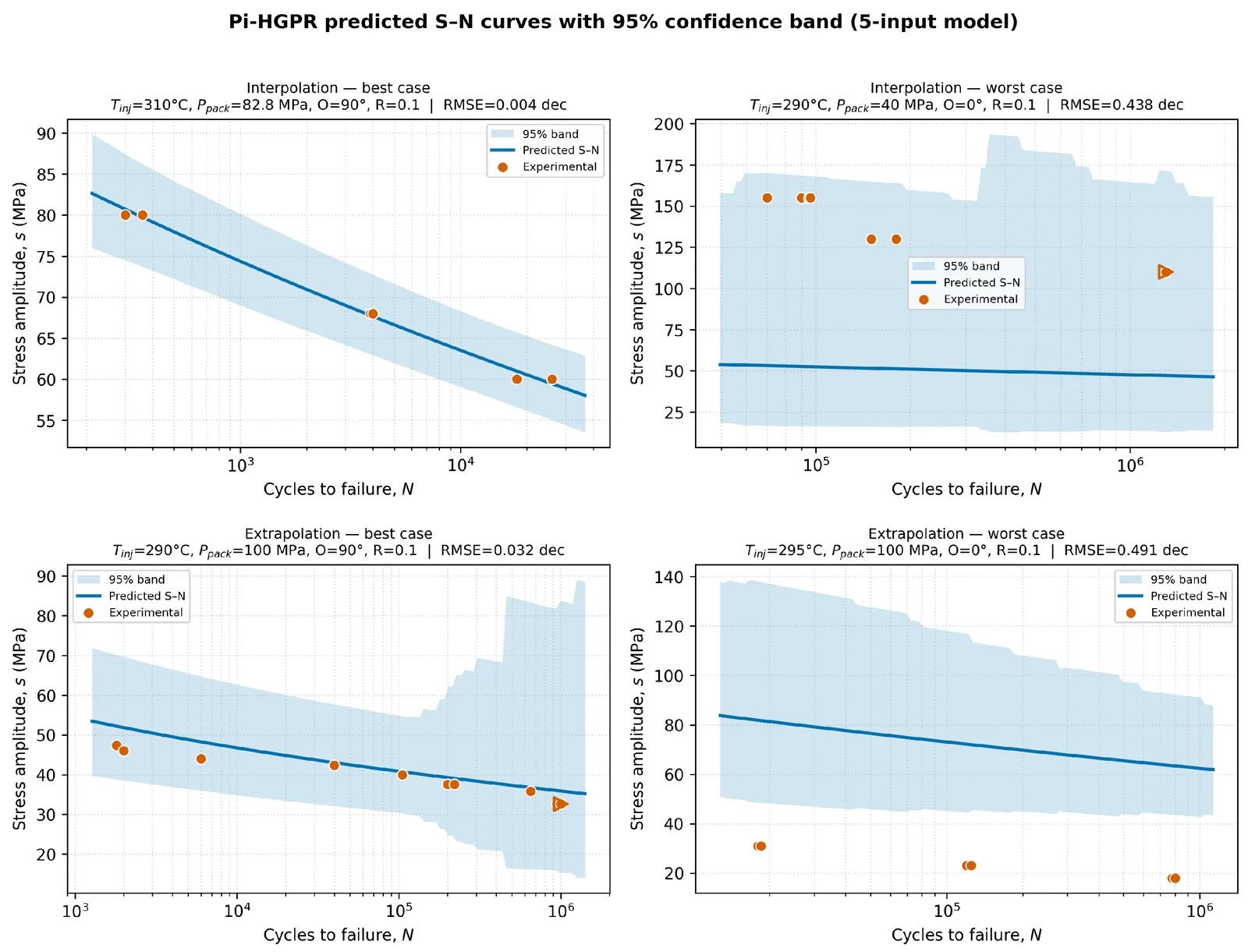
Predicted S–N curves (blue) with 95% prediction band for the best-, median- and worst-case conditions under each protocol, against the experimental data (orange; ▹ = runouts). The band widens where the data are scarce, while the worst extrapolation case illustrates the data-limited ceiling on generalization to unseen processing conditions. Top row: interpolation (point-wise split); bottom row: extrapolation (leave-one-out); left to right: best, median, and worst curve (curves with at least five points). Each panel is annotated with its processing condition and the out-of-fold RMSE in decades of $\log_{10}s$. A narrow band can be used directly as a design allowable; a wide band flags a condition outside the support of the data, for which a confirmatory test is required.

**Table 1:** Process parameters and testing conditions on each of the selected works.

	Tinjection	Ppacking	Stress Ratio R	Orientation	Testing Standard	Specimen Type	Test Frequency	Cite
PPGF	240	100	−1	0	ASTM D 638-03	dogbone	1	[17]
	220	40	−1	0	not specified	dogbone	4	[23]
	220	50	0.1	0	ASTM D7791-12	dogbone	5	[41]
	225	21	0.1, −1	0, 45, 90	ASTM 3479	dogbone	10	[42]
	230	58	−1	0	ASTM D790	rectangular	5	[43]
	220	40	0.1, −1	0, 90	not specified	dogbone	5	[44]
PA6GF	285	40	0.1	0	ASTM D7791-12	dogbone	5	[41]
	240	70	−1	0, 90	JIS K 7119	dognone	30	[45]
	261	96	0.1	0	not specified	dogbone	5	[46]
	300	75	0.1	0	not specified	dogbone	5	[47]
	295	100	0.1	0	not specified	dogbone	1	[48]
	290	40	0.1	0, 90	not specified	dogbone	4	[49]
	240	125	−1	0	not specified	gear	10	[50]
	290	100	0	0	ASTM D695-02a	dogbone	5	[51]
	290	40	0.1, −1	0	not specified	tubular	5	[52]
	310	82.8, 27.6, 55.2	0.1	0, 90	not specified	dogbone	1	[53]
	290	40	0.1	0	not specified	dogbone	5	[54]
	295	100	0.1	0	not specified	rectnagular	5	[55]
	275	45	0.1	0	not specified	notch rectangualar	4	[56]
PBTGF	260	50	0.1	0, 45, 90	not specified	dogbone	4	[3]
	260	80	−1, 0.1, 0.3	0, 90	ASTM D7791-12	dogbone	0.25–12	[57]
	250	65	0.1	0, 90	not specified	dogbone	5	[47]
	260	50	−1, 0.1	0	not specified	cilindrical dogbone	2	[58]
	260	80	−1	0	not specified	cilindrical dogbone	5	[59]

**Table 2:** Range of values in the dataset and reference.

Material	$T_{\mathrm{injection}}$ (°C)	$P_{\mathrm{packing}}$ (MPa)	Stress Ratio *R*	Orientation θ (°)	Reference
PPGF	220–280	21–100	−1–0.1	0, 45, 90	[17,23,41,42,43,44]
PA6GF	240–295	27.6–125	−1–0.1	0, 30, 90	[41,45,46,47,48,49,50,51,52,53,54,55,56]
PBTGF	250–260	50–80	−1–0.3	0, 45, 90	[3,47,57,58,59]

**Table 3:** Out-of-fold comparison of learning algorithms (target $\log_{10}s$, five inputs). Best value per column in bold.

	Interpolation		Extrapolation	
Model	$R^{2}$	MAPE (%)	$R^{2}$	MAPE (%)
Random Forest	0.790	19.3	0.521	35.4
Histogram Grad. Boosting	0.788	21.7	0.573	32.2
Pi-HGPR (this work)	**0.839**	**15.7**	**0.649**	**28.5**

**Table 4:** Ablation of the pipeline (cumulative). $R^{2}$ and MAPE on *s*; Cov. is the empirical coverage of the nominal 95% band.

	Interpolation			Extrapolation		
Configuration	$R^{2}$	MAPE	Cov.	$R^{2}$	MAPE	Cov.
Baseline GPR (zero mean, homosc.)	0.841	17.3	92	0.489	29.9	84
+ Basquin mean function	0.855	15.7	92	0.643	29.9	89
+ coupled hetero. filter (final)	0.839	15.7	93	0.649	28.5	86

**Table 5:** Out-of-fold indicators of the final Pi-HGPR model.

Indicator	Interpolation	Extrapolation
$R^{2}$ (on $\log_{10}s$)	0.839	0.649
RMSE (decades of $\log_{10}s$)	0.113	0.166
MAE (decades of $\log_{10}s$)	0.061	0.119
MAPE on *s* (%)	15.7	28.5
Within ±10% of *s* (%)	67	34
Within ±20% of *s* (%)	84	53
Coverage of the 95% band (%)	92.9	86.2

**Table 6:** Out-of-fold indicators of the final Pi-HGPR model disaggregated by specimen orientation and processing range (MAPE on *s*; Cov. is the empirical coverage of the nominal 95% prediction band).

				Interpolation		Extrapolation		
Variable	Group	Curves	Points	$R^{2}$	MAPE (%)	$R^{2}$	MAPE (%)	Cov. (%)
θ (°)	0	63	378	0.78	16.6	0.42	33.8	78
	30	2	17	– ^*a*^	6.4	– ^*a*^	5.4	100
	45	12	60	0.99	5.5	0.98	8.4	100
	90	39	220	0.80	17.7	0.73	26.7	95
$T_{\mathrm{injection}}$ (°C)	≤250	16	165	0.96	7.4	0.73	21.8	81
	251–280	73	296	0.75	22.6	0.63	28.8	91
	>280	27	214	0.67	12.6	0.04	33.3	84
$P_{\mathrm{packing}}$ (MPa)	≤45	22	223	0.90	9.7	0.66	30.0	76
	46–75	19	155	0.97	5.3	0.59	19.1	100
	>75	75	297	0.68	25.7	0.53	32.4	87

^*a*^ $R^{2}$ not reported for groups with fewer than five curves.

**Table 7:** ARD length-scales of the final model (standardized inputs; a shorter length-scale indicates a more relevant variable).

Input	Length-Scale *ℓ*	Relevance
$P_{\mathrm{packing}}$	0.49	Very high
$T_{\mathrm{injection}}$	0.58	High
Stress ratio *R*	2.18	Moderate
Specimen orientation θ	3.20	Moderate
$\log_{10}N$	8.21	Low (absorbed by the mean)

## Data Availability

The authors confirm that the data supporting the findings of this study are available within the article.
